# The Endocannabinoid System Activation as a Neural Network Desynchronizing Mediator for Seizure Suppression

**DOI:** 10.3389/fnbeh.2020.603245

**Published:** 2020-11-13

**Authors:** Daniel de Castro Medeiros, Vinícius Rosa Cota, Antonio Carlos P. Oliveira, Fabricio A. Moreira, Márcio Flávio Dutra Moraes

**Affiliations:** ^1^Núcleo de Neurociências, Departamento de Fisiologia e Biofísica, Instituto de Ciências Biológicas, Universidade Federal de Minas Gerais, Belo Horizonte, Brazil; ^2^Laboratório Interdisciplinar de Neuroengenharia e Neurociências, Departamento de Engenharia Elétrica, Universidade Federal de São João Del-Rei, São João Del-Rei, Brazil; ^3^Departamento de Farmacologia, Instituto de Ciências Biológicas, Universidade Federal de Minas Gerais, Belo Horizonte, Brazil; ^4^Centro de Tecnologia e Pesquisa em Magneto Ressonância, Programa de Pós-Graduação em Engenharia Elétrica, Universidade Federal de Minas Gerais, Belo Horizonte, Brazil

**Keywords:** epilepsy, cannabinoid system, neural synchrony oscillations, deep brain electrical stimulation, network decoupling, pharmacological treatment

## Abstract

The understanding that hyper-excitability and hyper-synchronism in epilepsy are indissociably bound by a cause-consequence relation has only recently been challenged. Thus, therapeutic strategies for seizure suppression have often aimed at inhibiting excitatory circuits and/or activating inhibitory ones. However, new approaches that aim to desynchronize networks or compromise abnormal coupling between adjacent neural circuitry have been proven effective, even at the cost of enhancing local neuronal activation. Although most of these novel perspectives targeting circuitry desynchronization and network coupling have been implemented by non-pharmacological devices, we argue that there may be endogenous neurochemical systems that act primarily in the desynchronization component of network behavior rather than dampening excitability of individual neurons. This review explores the endocannabinoid system as one such possible pharmacological landmark for mimicking a form of “on-demand” desynchronization analogous to those proposed by deep brain stimulation in the treatment of epilepsy. This essay discusses the evidence supporting the role of the endocannabinoid system in modulating the synchronization and/or coupling of distinct local neural circuitry; which presents obvious implications on the physiological setting of proper sensory-motor integration. Accordingly, the process of ictogenesis involves pathological circuit coupling that could be avoided, or at least have its spread throughout the containment of other areas, if such endogenous mechanisms of control could be activated or potentiated by pharmacological intervention. In addition, we will discuss evidence that supports not only a weaker role played on neuronal excitability but the potential of the endocannabinoid system strengthening its modulatory effect, only when circuitry coupling surpasses a level of activation.

## Introduction

Epilepsy is a severe brain disorder intimately associated with excessive neural excitability and synchrony whose treatment is still limited to a few pharmacological and non-pharmacological approaches (ketogenic diets, surgery; Devinsky et al., 2018; Loscher, 2020). The antiepileptic drugs primarily aim to reduce epileptic seizure occurrence by restraining the neuronal activity (reducing the excitatory or increasing the inhibitory transmission); thus, somehow, considering that network desynchronization would follow as a natural consequence of diminished excitability. Nevertheless, even with the substantial pharmacological arsenal available, drug treatment is still insufficient to ameliorate the symptoms and the course of the disease in some patients (refractory epilepsies; Perucca and Gilliam, 2012). Thus, new paradigms and strategies should be considered when approaching the neurobiology of epilepsy and developing new therapeutic interventions. This review will focus on the hypothesis that the endocannabinoid system can mediate epileptic seizure suppression by desynchronizing the neural networks rather than acting only at the excitation/inhibition balance.

### Cannabinoids and the Endocannabinoid System

The use of the herb *Cannabis sativa* (“marijuana”) for the treatment of epilepsy has been suggested for centuries (Zuardi, 2006). However, its clinical application was limited by its psychotropic effects, abuse liability, and the fact that its chemical composition remained unidentified until recently. Only in the second half of the twentieth century were its constituents, termed phytocannabinoids, finally characterized. Cannabis’s primary active substance is delta-9-tetrahydrocannabinol (THC; Mechoulam, 1970). However, various other compounds are interesting from a pharmacological standpoint, including cannabidiol (CBD), delta-9-tetrahydrocannabivarin, cannabidivarin, among others (Hill et al., 2013; Patra et al., 2019).

**FIGURE 1:**
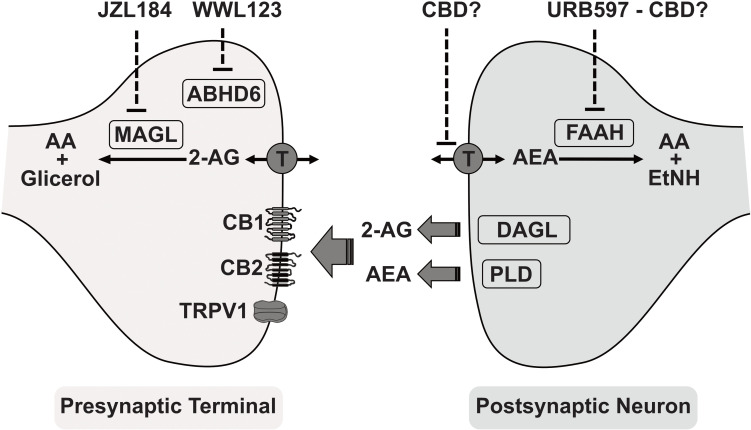
A simplified view of the endocannabinoid system and its main components. Endocannabinoids: Arachidonoylethanolamide (AEA, anandamide) and 2-arachidonoylglicerol (2-AG). Synthesizing enzymes: Diacylglycerol lipase (DAGL) and phospholipase-D (PLD). Membrane transporter (T). Cannabidiol (CBD) as inhibitor of anandamide reuptake and hydrolysis. Hydrolyzing enzymes: Monoacylglycerol lipase (MAGL), alpha/beta-Hydrolase domain containing 6 (ABHD6), and fatty acid amide hydrolase (FAAH). Enzymes inhibitors: JZL184 irreversible inhibitor for MAGL, URB597 relatively selective inhibitor of FAAH, WWL123 inhibitor of ABHD6. Receptors: cannabinoid type-1 (CB1), cannabinoid type-2 (CB2), and transient receptor potential vanilloid-1 (TRPV1). AEA metabolites: arachidonic acid (AA) and ethanolamide (EtNH). 2-AG metabolites: arachidonic acid (AA) and glicerol.

The chemical characterization of cannabis and THC isolation and synthesis has made it possible to obtain numerous synthetic derivatives (i.e., synthetic cannabinoids). The pharmacological studies with phytocannabinoids and synthetic cannabinoids finally led to the identification of their mechanisms of action and to the description of a new signaling mechanism in the brain, the endocannabinoid system (Pertwee et al., 2010; Figure 1). The endocannabinoid system comprises the Gi-coupled cannabinoid receptors CB_1_ and CB_2_ (the molecular targets of THC), the endogenous ligands (endocannabinoids) arachidonoyl ethanolamide (AEA, also anandamide) and 2-arachidonoylglycerol (2-AG), and the enzymes responsible for their metabolism. Endocannabinoids are proposed to function as a retrograde neurotransmission system, being produced from lipid membranes in postsynaptic neurons. Their actions are terminated after they are removed from the synaptic cleft by a membrane transporter and hydrolyzed in the intracellular medium (Pertwee et al., 2010; Mechoulam et al., 2014). The main enzymes responsible for metabolizing anandamide and 2-AG are fatty acid amide hydrolase (FAAH) and monoacylglycerol lipase (MAGL), respectively. Other enzymes also contribute to the biotransformation of endocannabinoids in the brain, such as cyclo-oxygenase 2 (COX-2), and alpha/beta-Hydrolase domain containing 6 (ABHD6). Additional receptors have also been described for endocannabinoids, among them the transient receptor potential vanilloid-1 channel (TRPV1), which can be activated by endogenous anandamide (Mechoulam et al., 2014).

In terms of clinical applications, THC and synthetic cannabinoids are of limited use as they can induce psychosis, abuse liability, amnesia, hyperphagia, and motor impairment. In fact, direct CB_1_ receptor agonists may even induce or aggravate epileptic seizures, depending on the dose (Asth et al., 2019). CBD, on the other hand, has been demonstrated to be efficacious in reducing epileptic seizures in both experimental and clinical settings (Billakota et al., 2019). Contrary to THC, CBD does not act as a CB_1_ receptor agonist; instead, its antiepileptic activity seems to occur by inhibiting anandamide reuptake and hydrolysis, increasing the brain levels of this endocannabinoid and thereby activating CB_1_ receptor signaling (Vilela et al., 2013). Accordingly, CBD antiepileptic effects depend on the PI3K/mTOR intracellular pathway, a signal transduction mechanism coupled to the CB_1_ receptor (Gobira et al., 2015; Lima et al., 2020). Other phytocannabinoids have been reported as potential antiepileptic drugs, among them, cannabidivarin, and delta-9-cannabivarin (Hill et al., 2010, 2013).

#### Cannabinoid on-Demand and Circuit Breaker Functions

The detailed understanding of the physiological aspects of the endocannabinoid system has offered new pharmacological possibilities beyond the phytocannabinoids. Evidence converging from various experimental approaches suggest that endocannabinoid synthesis can be triggered by post-synaptic neurons in response to calcium influx after excessive glutamate release and neuronal excitability. Once in the synaptic cleft, they bind to presynaptic CB_1_ receptors whose activation restrains hyperexcitability and attenuate neurotransmitter release, therefore working as a negative feedback mechanism modulating synaptic transmission (Maejima et al., 2001; Wilson and Nicoll, 2001). Remarkably, seizure-inducing substances increase anandamide levels in the hippocampus and have their effect magnified by CB_1_ receptor blockade (Marsicano et al., 2003; Wallace et al., 2003). Based on these observations, endocannabinoids have been proposed to function as an on-demand mechanism protecting the brain against hyperexcitability and activity-dependent excitotoxicity (Marsicano et al., 2003). The molecular mechanisms at the synaptic levels possibly entail presynaptic glutamate release followed by calcium-triggered endocannabinoid synthesis and release from the post-synaptic terminal; endocannabinoids activate presynaptic CB_1_ receptor, which activates a Gi-protein and triggers an intracellular cascade whose consequence is a reduction in calcium influx and glutamate release. Altogether, this mechanism would work as a synaptic circuit breaker (Katona and Freund, 2008; Katona, 2015; Soltesz et al., 2015).

These unique characteristics point to the endocannabinoid system as an attractive target for pharmacological intervention for the treatment of epilepsies. Theoretically, the selective inhibition of endocannabinoid-hydrolyzing enzymes could work with anatomical and temporal resolution, restraining synaptic activity only under circumstances in which excessive activity (excitotoxicity) would occur. In line with this hypothesis, synthetic compounds that inhibit the enzymes responsible for degrading anandamide and 2-AG yields favorable results in experimental models of seizure and epilepsy. Concerning anandamide hydrolysis, the selective FAAH inhibitor AM374 inhibits kainic acid-induced seizure and neurotoxicity (Karanian et al., 2007). Moreover, the FAAH inhibitor URB597 increases the threshold of pentylenetetrazole-induced behavioral and electroencephalographic seizures (Vilela et al., 2013). As for the 2-AG-related enzymes, ABHD-6 inhibition also reduces PTZ-induced seizures (Naydenov et al., 2014), whereas MAGL inhibition delays the consequences of kindling induced by electrical stimulation (ES) of the amygdala (von Rüden et al., 2015). Importantly, endocannabinoid hydrolysis inhibitors tend to have a safer pharmacological profile as compared to direct CB_1_ agonists, as they seem less prone to induce psychosis, motor impairment, and addiction, which can be attributed to the on-demand functioning of endocannabinoids (Asth et al., 2019).

Therefore, the endocannabinoid receptors and molecules deal with the brain hyperexcitability, one crucial aspect of epilepsy, by modulating the synaptic transmission in a neural activity-dependent manner. Nevertheless, recent studies have demonstrated that the cannabinoid system also modifies the neural synchrony, in some cases with a marginal effect on overall excitability, which is intimately associated with complex brain functions (e.g., sensation, perception, and cognition) and neurological disorders as schizophrenia, Alzheimer’s disease, and epilepsy (Uhlhaas and Singer, 2006).

### Epilepsy as a Network Dysfunction and Hypersynchronous Disease

Although synchronization and hypersynchronization are largely used to describe neural phenomena in general and epilepsy in particular, these terms are somehow loosely defined in the literature. In a system containing multiple oscillating subsystems such as the brain, synchronism can be described as a driving influence of an oscillator toward another one (Jensen and Colgin, 2007). This means that objective dynamical descriptors (e.g., amplitude, phase, and frequency, etc.) will display a mathematical relation of the kind *y* = *f*(*x*) between oscillations if they are synchronized. Given the timescale of neural events of interest (in the order of milliseconds), electrographic recordings such as scalp electroencephalogram (EEG) in humans or intracranial local field potentials (LFP) in experimental animals are the main choice for objectively assessing neurodynamical synchronism underlying brain function and disease. Myriad approaches have been used to perform such investigation of EEG and LFP signals, ranging from assessment of occurrence and temporal coincidence of meaningful electrographic signatures by visual inspection to advanced computerized mathematical analyses such as cross-correlation, coherence, partial directed coherence, Granger causality, mutual information, phase lock value, and cross-frequency phase-amplitude coupling (CFC; Quian Quiroga et al., 2002; Kreuz et al., 2007).

In this perspective, while normal levels of synchronism between neural structures underlie brain function (Schnitzler and Gross, 2005; Womelsdorf et al., 2007), aberrations lead to dysfunction (Uhlhaas and Singer, 2006). For instance, it is now well-established that consolidation of declarative memory largely relies on triple phase-amplitude coupling between cortico-cortical slow oscillations, thalamocortical spindles, and hippocampal ripples across the sleep-wake cycle (Klinzing et al., 2019). In contrast, epilepsy is understood as a disease of hypersynchronization; a rationale supported by an ever-increasing number of experimental observations. Starting from the occurrence of highly-synchronous paroxysms such as epileptiform polyspikes (Wu et al., 2013), hypersynchronization can also be evidenced by the temporal and spatial pattern of spread of aberrant activity across nodes of ictogenic networks involving the hippocampus, amygdala, and parahippocampal areas in Temporal Lobe Epilepsy (TLE) and also midbrain and hindbrain structures in generalized tonic-clonic seizures (de Curtis and Avanzini, 2001; Avoli et al., 2002; de Guzman et al., 2004; Moraes et al., 2005a; Bertram, 2013). In the same vein, modifications in the expression of electrographic activity induced by manipulations of neural circuitry (lesions and transections) are additional proof of network synchronization underlying epileptic phenomena (Imamura et al., 2001; Moraes et al., 2005b). Finally, increases in phase-amplitude CFC between different pairs of band frequencies (Nariai et al., 2011; Guirgis et al., 2013; Edakawa et al., 2016) and *in silico* findings from non-linear dynamics analysis (Kalitzin et al., 2019) further corroborate this view. It is important to highlight, though, that hypersynchronization is not ubiquitous during ictogenesis, and there has also been evidence of desynchronization, at least in specific areas, frequencies, and time points of the process (Netoff and Schiff, 2002; Jiruska et al., 2013).

Not only epileptic phenomena have been quantitatively studied by this measure, but also novel therapeutic interventions (pharmacological or not) are screened according to their effects on synchronization levels of brain signals. For instance, different modalities of Deep Brain Stimulation (DBS) have been found, among other effects, to suppress aberrant oscillations while inducing beneficial rhythms (Udupa and Chen, 2015). In fact, the DBS delivered by a responsive neurostimulation system (RNS^TM^ System, NeuroPace, Inc.) to patients with epilepsy acutely suppressed gamma frequency (35–100 Hz) phase-locking (Sohal and Sun, 2011). Using eigenvalue dynamics computed over cross-correlation matrices, Schindler and colleagues (2007) have also found that EEG synchronization levels depend on parameter settings of low-frequency stimulation of the seizure onset zone in humans (Schindler et al., 2007).

Of particular interest here, some ES approaches have been tailored to specifically tackle synchronization as a means to treat epilepsy in further corroboration of the notion of anticonvulsant effects of desynchronization. A non-standard form of low-frequency stimulation (four pulses per second in average) with randomized intervals between pulses, termed non-periodic stimulation (NPS) and devised by our group, has been shown to effectively suppress acute seizures induced by PTZ (Cota et al., 2009) and in chronic seizures induced in the late phase of the pilocarpine model of TLE (de Oliveira et al., 2014). Electrographically, NPS has been shown to rectify spectral signatures (de Souza Silva et al., 2019) and possibly to decrease the duration of epileptiform activity, the number and the frequency of epileptiform spikes (de Oliveira et al., 2019). An approach very similar to NPS termed Temporally Irregular DBS (TiDBS) has been used to effectively impair epileptogenesis induced by amygdalar kindling, shortening daily afterdischarge duration, and interfering with propagation patterns of epileptiform activity (Santos-Valencia et al., 2019). Other forms of desynchronizing ES have been used to suppress seizures or decrease cortical excitability, with correlated electrographical findings (Quinkert et al., 2010; Wyckhuys et al., 2010). In fact, the temporal pattern of ES is now considered to have a central role in the modulation of neuronal activity (Zheng et al., 2020) and to suppress aberrant synchronization in epilepsy and many other neurological disorders (Grill, 2018).

From this set of findings, one can conclude that assessing complex epileptic phenomena in the network level alongside its emerging properties such as synchronization (Garcia-Cairasco, 2009) may represent not only a fruitful approach to understand the pathophysiology of epilepsy, but also to develop novel treatment (pharmacological or not) in an engineered and thus efficacious way (Sunderam et al., 2010). This is exactly the venue this review explores, associating the on-demand endocannabinoid system and pharmacological targets to its ability to modulate coupling among distinct network oscillators without necessarily dampening individual neuronal activity itself. This framework is further explored in the following sections.

### Endocannabinoid System Diminishes the Neural Organization

Fluctuations in the electrical field potential (LFP) are permanently present at the cerebral extracellular medium, reflecting the alternating pockets of higher/lower recruitment probability of localized population of neurons (Buzsáki et al., 2012). The pace activity offers a temporal-organized framework for neural communication (Fries, 2005; Buzsáki, 2010), and both local and distant neuronal ensembles (task-demanding cells that fire in a constricted window) transiently synchronize the oscillatory activity during information processing (O’Keefe and Recce, 1993; Varela et al., 2001; Bosman et al., 2012). Disturbance in the fine-tuning of the network time-coupling [mainly regulated by inhibitory synapses (Buzsáki and Chrobak, 1995; Whittington et al., 2000)] is associated with cognitive disorders and neurological pathologies (Uhlhaas and Singer, 2012). Important to note that the cannabinoid receptors are the most abundant G protein-coupled receptor in the brain and present at GABAergic and glutamatergic axon terminals (Devane et al., 1988), but up to ten times more prevalent at the former (Kawamura et al., 2006). Hence, alterations at the cannabinoid system may potentially perturb the neural connections and, consequently, the coupling and generation of oscillatory patterns related to physiological functions.

The LFP brain oscillations range from very slow (<0.01 Hz) to ultrafast frequencies (200–600 Hz), and distinct band rhythms become prominent when cerebral structures engage in specific tasks (Buzsáki and Draguhn, 2004). Perceptual functions are closely related to gamma oscillations (30–80 Hz) and involve timed interaction of distributed neural groups (Fries, 2005, 2009). Even though gamma rhythm typically emerges from local networks, its remote synchronization can be performed by long-distant neurons and by the interaction with slower frequencies that modulate activity over extensive spatial regions (Buzsáki et al., 2013), i.e., phase coding. Gamma synchrony is considered an essential mechanism for binding sensory features in sparse structures, an element present in consciousness, and modifications in this rhythm may underline perceptual disturbances seen in psychosis (Fries, 2005, 2009; Uhlhaas and Singer, 2006; Wang, 2010). Schizophrenic subjects present deficits in the perceptual organization, correlated with the reduction of gamma power and synchrony over distributed areas (Uhlhaas et al., 2006). Similarly, healthy humans administered with CB1R agonist (THC) exhibit psychosis-relevant effects associated with gamma oscillation disorder (coherence reduction during auditory evoked response test; Cortes-Briones et al., 2015). *In vitro* and *in vivo* animal investigations additionally demonstrated that CB1-agonist disturbs gamma rhythm in limbic system areas (reduction synchrony and power, respectively) and impair auditory processing (sensory gating), acting mainly in GABAergic synapses (Hájos et al., 2000, Hajós et al., 2008). Nonetheless, the disturbance induced by cannabinoids expands beyond gamma and perceptual functions, also affecting low frequency generation, phase-coding and other cognitive faculties.

Compared with faster frequencies, slow oscillatory rhythms are associated with a more extensive brain volume alteration, longer time-window discharging probability and, the integration of a significant higher number of neurons (von Stein and Sarnthein, 2000; Quilichini et al., 2010; Buzsáki and Wang, 2012). Of particular interest to phenomena involving the function/dysfunction of the hippocampus (i.e., memory/TLE, respectively), the slow frequency theta rhythm (4–12 Hz) is related to the temporal organization of faster frequencies (e.g., gamma-band by cross-frequency coupling) and the coordination of local and distant unit-firing (Mizuseki et al., 2009; Quilichini et al., 2010; Lisman and Jensen, 2013). Human and animal studies have shown the effect of potentiating the cannabinoid system in theta oscillations disruption and memory impairment. Morrison et al. demonstrated that healthy patients administered with CB1 agonist (THC) performed poorly at working-memory tests and presented a reduction of theta power and coherence in frontal lobe electrodes. Importantly, the disrupting of network dynamics, revealed by coherence diminishing, correlated with positive psychotic symptoms (Morrison et al., 2011). CB1R activation (CP55940-potent agonist) also disturbed the synchrony of the rats’ neural oscillations at the medial prefrontal cortex (mPFC) and hippocampus during end-to-end T-maze spatial working memory task. In addition, animals presented power reduction in the gamma-band at the mPFC and in the theta-band at the hippocampus, decreasing the theta coherence between hippocampus-mPFC. This work also showed a substantial compromise of the prefrontal unit phase-locking activity to the hippocampus theta rhythm, which correlates to reduced cognitive performance (Kucewicz et al., 2011). Robe et al. also demonstrated that the CB1 agonist CP55940 decreased hippocampal theta power (in freely moving rats) associated with memory impairment. Interestingly, the activation of CB1R occasioned a severe disruption of cell assembly time coordination. However, there was no spatial remap of the place cells and only a marginal reduction in fire rate and no correlation with LFP power change. The authors argued that the CB1 dyssynchrony-effect might be the origin of theta power reduction, since the excitatory/inhibitory firing rate balance was minimally affected (Robbe et al., 2006; Robbe and Buzsáki, 2009). If this claim is valid, the CB1-induced disruption of the time organization may play a far more important role at the network synchrony than on overall network excitability – which would explain behavioral disturbances and cognitive impairment. Additionally, it could contribute to the LFP power decrease seen in several structures, as described previously.

Despite the rhythm disordering in healthy subjects, the CB activation may be beneficial to neural networks prone to develop hypersynchronous state. The synchrony reduction caused by CBR1 could balance the abnormal coupling among microcircuits present in epileptic brains, decreasing the pathological oscillatory attractor-effect, and, consequently, the occurrence of the seizures. Important to note, the endocannabinoid system offers an “on-demand” approach, with a major effect in excessively active neural ensembles [higher coupling probability (Kudela et al., 2003)], which is quite suitable for long-term treatments.

### Cannabinoid Activity on Inhibitory Cells as a Mechanism for Neural Network Desynchrony and Seizure Suppression

**FIGURE 2:**
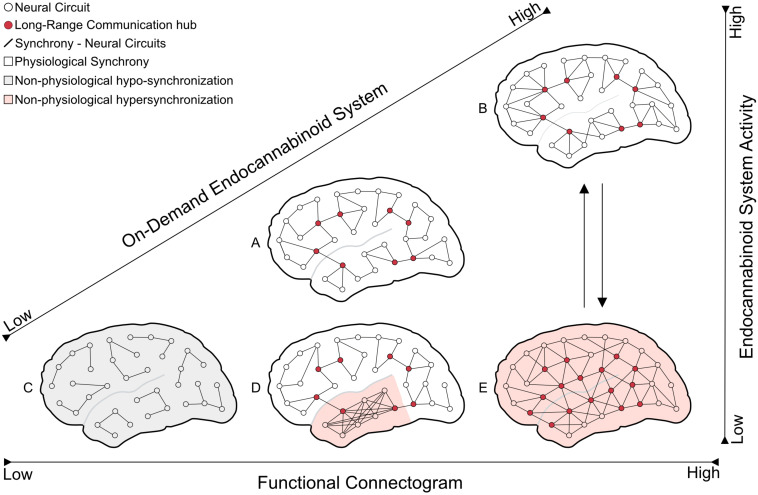
The schematic model depicts the endocannabinoid system’s hypothetical effect on network synchrony at different functional connectogram levels. The on-demand endocannabinoid system activity would suppress the pathological excessive neural synchrony (seizure) and maintain the brain in physiological conditions even in functional connectogram fluctuations. **(A)** brain in the physiological resting state; **(B)** brain in the physiological state of a hard mental workload (increasing of neural network synchronization compared with A); **(C)** brain at anesthetized/comatose state (severe decrease in circuits communication – hypo-synchronization); **(D)** disordered/excessive neural synchrony at a distinct brain area (focal seizure – hypersynchronization); and **(E)** extensive and unspecific pathological synchronization (generalized seizure – hypersynchronization).

Although pertaining to the same neurochemical system, targeting directly CB1 receptors or the anabolic/catabolic pathways of endocannabinoid metabolism are fundamentally very different approaches. The most noticeable particularity of this specific neurochemical system, as shown in previous sections of this review, is its ability to promote homeostatic modulation of synaptic activity by targeting the presynaptic neuron, through a feedback mechanism. And, such backward modulation is mostly triggered by “abnormal” or excessive postsynaptic activation, that, consequently, promotes increased endocannabinoid release aimed at presynaptic receptors (Figure 2). It should be clear that such “on-demand” recruitment of synaptic homeostatic modulation would be lost if pharmacological agents were to target the receptors directly [for review see Katona and Freund (2008)].

The quasi-specific co-expression of CB1 receptors in CCK GABAergic interneurons may help explain the endocannabinoid system’s prominent role in regulating coupling-strength between neuronal oscillators rather than on the hippocampal network excitability itself (Katona et al., 1999). Evidence shows that parvalbumin-positive GABAergic interneurons (PV^+^) are fast-spiking, create time-delimited pockets of oscillations – sometimes referred to as the hippocampal “clocks,” but are also involved in very strong lateral inhibition modulation of similar feedforward/feedback microcircuit motifs within the hippocampus. Altogether, PV^+^ seems to promote an efficient mechanism of pattern separation with these circuit motifs that are consistent with engram formation and discrimination associated with different memory traces (Espinoza et al., 2018). On the other hand, the role of CCK positive interneurons seems much less specific – as well as less known. The CCK^+^ are slower firing interneurons (Klausberger et al., 2005), with a lesser strict set of connectivity rules within the hippocampus and have been suggested to modulate much more complex behavior traits (e.g., mood regulation), that are certainly dependent on the temporal and spatial organization of multiple engrams (Freund, 2003). Thus, the endocannabinoid system may play an important part in how spatial pockets of hippocampal microcircuit patterns interact with each other in time, a hypothesis that can extend for other neural areas (Iball and Ali, 2011). In fact, if such a claim were true, one would expect the pharmacological manipulation of the cannabinoidergic system to affect slower oscillations, associated with the temporal arrangement of hippocampal microcircuit motifs (Robbe et al., 2006; Robbe and Buzsáki, 2009), to a much greater degree than the faster oscillators (associated to a more local or specific circuit motifs – e.g., fast gamma oscillations).

The loss or “silencing” of GABAergic interneurons are known to play an important role in TLE (McNamara, 1994; Zhang and Buckmaster, 2009). In fact, it has been suggested that the circuit rearrangement promotes the sustained epileptiform activity by compromising inhibitory feedback/feedforward microcircuits in hippocampal networks (Paz and Huguenard, 2015). Even under physiological conditions, considering the untampered hippocampal circuitry, rhythm generation is known to be highly dependent on GABAergic interneurons (Cobb et al., 1995; Buzsáki and Wang, 2012). The Medial Septal (MS) neurons projecting to hippocampal GABAergic interneurons and its ability to coordinate the firing patterns of specific circuit motifs generating GAMA activity has been proven essential to produce theta wave oscillations (Dragoi et al., 1999; Hangya et al., 2009). In addition, the temporal and spatial organization of multiple engrams has a strict phase correlation with the overall theta oscillation, rather than with fixed time delays between the local oscillators themselves (Petersen and Buzsáki, 2020). Thus, if one interprets the ictogenic process as several microcircuits being coupled together, throughout massive amounts of neural tissue, with complete disregard to a specific patterns associated to a memory trace, or traces presented in sequence; the pathophysiological counterpart would be that the system responsible for circuit discrimination and organization must have been compromised. Indeed, there is a selective loss of CCK^+^ interneurons in TLE (Wyeth et al., 2010), theta oscillations are much more compromised than gamma oscillations (Inostroza et al., 2013), MS GABAergic interneurons project to CCK^+^ interneurons (although not exclusively; Freund and Antal, 1988; Unal et al., 2015), and, as mentioned before, express endocannabinoid receptors. Altogether, CCK^+^ interneurons seem to play an important role in synchronizing and differentiating the microcircuits composed of localized groups of hippocampal pyramidal cells and, when compromised, unleash PV + interneurons to synchronize the entire network.

Therefore, some of the same mechanisms associated with the behavioral manifestations after the recreational use of *Cannabis sativa*, might explain its success in treating patients with epilepsy. The same synchrony disturbance, or disorganization of microcircuit synchronous recruitment, that would make a subject under the influence to express disconnected phrases and ideas, would be very beneficial to disrupt an “abnormal attractor” coupling a massive group of pyramidal cell discharges that exist in epilepsy. In point of fact, it would be even better if such a disruption would occur only when absolutely needed, i.e., “on-demand.” That is obviously the case of asynchronous electrical stimulation triggered by abnormal ictal activity and (closed-loop), and, as suggested by this review, the potentiation of the endocannabinoid system could render the same effect as an independent pharmacological treatment.

## Conclusion

The NPS – DBS and the endocannabinoid pharmacological therapeutic approaches are obviously quite different treatment strategies for epilepsy, with no evidence in the literature of reciprocal modulation. Nevertheless, as proposed by this review, both strategies may share the common goal of focusing on desynchronizing network activity without necessarily affecting excitation/inhibition balance (Medeiros and Moraes, 2014). It is quite important to clarify the fact that the strategies are not mutually exclusive and may very well have a synergetic effect if considered as a form of polytherapy. In addition, NPS – DBS could also benefit from exploratory probing stimulation, to test for abnormal network coupling, conferring an “on-demand” characteristic to its presentation (Medeiros et al., 2014). Altogether, both strategies would be complementary in the sense that on-demand-DBS would have a much faster action, with a narrower time-window constant, while endocannabinoid targeting would present long-term background action on network hypersynchronization.

More research and experimental data are needed in order to determine if DBS therapy (time-fixed pulses or NPS) predominantly has its effect by modulating or recruiting the endocannabinoid system; which is, at this time, speculative and solely based on the possible coincidental mechanisms of both therapeutical approaches. Aside from this potential caveat, a hypothetical rationale is that particular DBS patterns (high-frequency stimulation – over 50 Hz, or short NPS interpulse lengths – below 20 ms) could recruit the on-demand release of endogenous CBR1 ligands triggered by the increase in abnormal neural activity/connectivity. Thus, the DBS would directly modulate the brain’s dynamic functional connectogram by recruiting a built-in on-demand “circuit-breaker system” (i.e., the endocannabinoid system), consequently disrupting neural network abnormal synchronization. According to this proposal, the DBS and cannabinoid system would have a bidirectional collaborative effect on seizure suppression, substantially enhancing the common outcomes.

## Author Contributions

DM, FM, and MM conceived the presented idea and supervised the project. DM, VC, FM, and MM wrote the manuscript. AO provided critical feedback and helped shape the manuscript. All authors reviewed, edited, and approved the manuscript.

## Conflict of Interest

The authors declare that the research was conducted in the absence of any commercial or financial relationships that could be construed as a potential conflict of interest.
